# Convergence in mycorrhizal fungal communities due to drought, plant competition, parasitism, and susceptibility to herbivory: consequences for fungi and host plants

**DOI:** 10.3389/fmicb.2014.00306

**Published:** 2014-06-25

**Authors:** Catherine A. Gehring, Rebecca C. Mueller, Kristin E. Haskins, Tine K. Rubow, Thomas G. Whitham

**Affiliations:** ^1^Department of Biological Sciences and Merriam Powell Center for Environmental Research, Northern Arizona UniversityFlagstaff, AZ, USA; ^2^Department of Biological Sciences, Northern Arizona University, Flagstaff, AZ, USA

**Keywords:** climate change, community convergence, community disassembly, competition, drought, ectomycorrhizal fungi, herbivory, mistletoe parasitism

## Abstract

Plants and mycorrhizal fungi influence each other’s abundance, diversity, and distribution. How other biotic interactions affect the mycorrhizal symbiosis is less well understood. Likewise, we know little about the effects of climate change on the fungal component of the symbiosis or its function. We synthesized our long-term studies on the influence of plant parasites, insect herbivores, competing trees, and drought on the ectomycorrhizal fungal communities associated with a foundation tree species of the southwestern United States, pinyon pine (*Pinus edulis*), and described how these changes feed back to affect host plant performance. We found that drought and all three of the biotic interactions studied resulted in similar shifts in ectomycorrhizal fungal community composition, demonstrating a convergence of the community towards dominance by a few closely related fungal taxa. Ectomycorrhizal fungi responded similarly to each of these stressors resulting in a predictable trajectory of community disassembly, consistent with ecological theory. Although we predicted that the fungal communities associated with trees stressed by drought, herbivory, competition, and parasitism would be poor mutualists, we found the opposite pattern in field studies. Our results suggest that climate change and the increased importance of herbivores, competitors, and parasites that can be associated with it, may ultimately lead to reductions in ectomycorrhizal fungal diversity, but that the remaining fungal community may be beneficial to host trees under the current climate and the warmer, drier climate predicted for the future.

## INTRODUCTION

The aims of the field of community ecology include understanding how communities respond to changing environmental conditions, as well as, the consequences of those changes for both communities and ecosystems. Understanding community trajectories is currently of greater importance due to agents of rapid environmental change such as climate warming and the introduction of non-native species (Tylianakis et al., 2008). Both of these global changes can alter interspecific interactions with consequences for species persistence and biodiversity (Voigt et al., 2003; Zarnetske et al., 2012). Changes in herbivore and predator communities due to global change have been argued to have disproportionate effects on the broader community (Zarnetske et al., 2012). In grassland, warmer temperatures and higher nitrogen increased insect herbivore biomass, with no concomitant increase in parasitoids (de Sassi and Tylianakis, 2012). Global change impacts on mutualist communities are predicted to be among the most extreme (Dunn et al., 2009). For example, disruption of vertebrate seed dispersal mutualisms may create “widow” species that lack mutualist services (Aslan et al., 2013). Likewise, disruption of mutualistic associations between plants and mycorrhizal fungi by non-native plant species may tilt the competitive balance towards non-native plants (Vogelsang and Bever, 2009; Meinhardt and Gehring, 2012).

While there are abundant examples of the impacts of environmental change on communities, studies are often necessarily focused on one aspect of environmental change, leaving us with little information on the similarities or differences in community trajectories in response to different types of environmental change. For example, a significant body of research has demonstrated the importance of the symbiosis between plants and mycorrhizal fungi at the individual, population, community, and ecosystem level (see examples in Johnson and Gehring, 2007; Smith and Read, 2008), and several individual studies have documented shifts in fungal communities due to environmental changes such as nitrogen deposition and climate change (e.g., (Lilleskov et al., 2002; Heinemeyer et al., 2004). However, many of these studies have focused on the relationships between plants and fungi in isolation from other biotic interactions such as competition, facilitation, and herbivory, and even fewer studies have determined if fungal communities respond similarly to varied perturbations. Do mycorrhizal fungi respond similarly to the parasites and herbivores that feed on their host plants, for example? Do communities change in similar ways if the stressor is abiotic versus biotic? The consequences of fungal community changes for host plant growth and survival are often also poorly known, but fungal symbionts, including mycorrhizal fungi, may alter host plant response to global change (Kivlin et al., 2013). Understanding the feedbacks among global changes, mycorrhizal fungal communities and host plant survival will provide insights into the long-term effects of global change on ecosystems.

In this paper, we examined the interactions between communities of ectomycorrhizal fungi (EMF) associated with a single plant species as it interacted with an insect herbivore (the scale insect, *Matsucoccus acalyptus* Herman), a plant parasite (dwarf mistletoe, *Arceuthobium divaricatum* Engelm), and an interspecific belowground tree competitor (*Juniperus monosperma* Engelm). We also examined if abiotic and biotic stressors resulted in similar community shifts by comparing the EMF of insect herbivore-affected and unaffected trees at two time points, one prior to long-term drought in the study area and one in the midst of a severe, ongoing drought that began in 1995 (Mueller et al., 2005). We focused on EMF because of the intimate trading partnership they develop with their plant hosts in which soil resources (nutrients and water) are exchanged for photosynthate (Smith and Read, 2008). Stressors such as drought, herbivory, parasitism, and competition all may increase host plant need for soil resources while potentially reducing the ability of the plant to provide photosynthate to EMF whose carbon requirements can be substantial (Nehls, 2008). EMF also represent good models for community studies because they are diverse, with an estimated 200+ genera from eleven orders involved in the association (Tedersoo et al., 2010), and their communities can be highly responsive to environmental change (Swaty et al., 2004). We tested the following hypotheses: (1) Communities of EMF will respond similarly to biotic stresses of parasitism, herbivory, and competition leading to a convergence in community structure associated with biotic stress. We predicted that these biotic stresses would result in similar changes in EMF community composition because they likely alter the ability of host plants to provide photosynthate to EMF, resulting in an EMF community composed of species with lower carbon demands. (2) Communities of EMF will respond similarly to the abiotic stress of drought as they do to the biotic stress of herbivory. Again, we reasoned that chronic herbivory and drought stress would affect EMF communities similarly because both stressors were likely to lead to photosynthate limitation. (3) Plants colonized by the EMF community associated with high herbivory, parasitism, and competition will exhibit poor growth. Previous studies have shown that EMF with low carbon requirements tend to invest less in structures such as external hyphae (Saikkonen et al., 1999), suggesting that they may be inferior mutualists.

We tested these hypotheses using DNA sequence data on the root colonizing EMF communities associated with pinyon pine (*Pinus edulis* Engelm.), a foundation tree species distributed across large areas of the southwestern US. This species has experienced substantial, drought related mortality in recent years across much of its distribution (Mueller et al., 2005; Garrity et al., 2013). We have previously shown that herbivory by a needle feeding scale insect (Gehring and Whitham, 2002), parasitism by a dwarf mistletoe (Mueller and Gehring, 2006), and competition with co-dominant juniper (Haskins and Gehring, 2004) altered EMF community composition. Here we synthesized these data sets and conducted new analyses to determine if these varying biotic stressors had similar impacts on EMF community composition (Hypothesis 1). Repeated sampling of the same herbivore resistant and herbivore susceptible trees before and during drought allowed us to assess the similarity of drought and herbivore affected communities (Hypothesis 2). Long-term herbivore removal experiments provided us with the opportunity to examine the influence of changes in EMF community composition on plant growth when the direct impact of herbivores on plant performance was dramatically reduced (Hypothesis 3). This study is important because it compares the responses of EMF communities to different types of stressors, both biotic and abiotic, and examines the potential consequences of community changes to the host plant. Studies of such complex interactions are of growing importance given that global change has been shown to influence nearly every type of species interaction (Tylianakis et al., 2008). Also, while community disassembly, the nonrandom process of progressive species decline or loss, has been demonstrated in response to a variety of global changes (Zavaleta et al., 2009), the dynamics of this process in the mycorrhizal symbiosis remains poorly understood.

## MATERIALS AND METHODS

### HYPOTHESIS 1: COMPETITION, PARASITISM, AND HERBIVORY WILL HAVE SIMILAR EFFECTS ON EMF COMMUNITY COMPOSITION

To test Hypothesis 1, we used previously published data on the EMF communities of *P. edulis* that experienced low versus high levels of three types of negative biological interactions: (1) parasitism by the dwarf mistletoe (*A. divaricatum*) which derives water, mineral nutrients, and a portion of its carbon requirements from its host plant (Mueller and Gehring, 2006), (2) belowground competition with juniper (*J. monosperma*), a co-dominant, drought tolerant tree in the pinyon-juniper woodland ecosystem (Haskins and Gehring, 2004), and (3) herbivory by a scale insect (*M. acalyptus*) that feeds on the leaf mesophyll tissue of juvenile *P. edulis* leading to premature needle abscission, reduced growth, and a characteristic poodle tail architecture of susceptible trees (Cobb and Whitham, 1993), whereas resistant trees have normal tree architecture and a full complement of needle cohorts. Results of scale insect transfer experiments suggested that resistance versus susceptibility to the scale is genetically based (Cobb and Whitham, 1993; Gehring et al., 1997). Although species richness and diversity did not respond consistently across studies, in all cases the EMF community composition of *P. edulis* experiencing low levels of the biotic interaction were significantly different from those experiencing high levels of the biotic interaction (Gehring and Whitham, 2002; Haskins and Gehring, 2004; Mueller and Gehring, 2006). In the case of *M. acalyptus*, degree of foliage loss due to scale herbivory on scale resistant and susceptible trees was significantly, linearly associated with degree of change in EMF community composition (*r*^2^ = 0.591, *P* < 0.001; Gehring and Bennett, 2009). We took advantage of natural variation in herbivory and mistletoe parasitism but experimentally reduced belowground competition with juniper by trenching. In this study, we compared the community composition of EMF across the studies to determine if these three biotic stressors resulted in convergent or divergent communities.

Although detailed methods can be found in the individual publications, a brief description follows. All studies were conducted in pinyon-juniper woodlands in northern Arizona, but soil type, year of sampling, and tree size and age varied among studies. Within a study, all high and low biotic interaction trees were intermixed at the same site, but sites differed among studies. Trees experiencing competition and insect herbivory occurred within 2 km of one another on nutrient poor volcanic soils, but mature trees were sampled in the competition study and juvenile trees (pre-reproduction) in the herbivory study. Plant parasite effects on *P. edulis* EMF communities were studied on mature trees at sites with better developed soils of volcanic origin more than 35 km distant from the other sites. Because of this variation in sites, tree age, and year of sampling, and the high diversity of EMF, we expected that trees experiencing low levels of these different negative biotic interactions would have different communities.

We used similar methods to characterize EMF communities. Briefly, we collected fine roots (<2 mm diameter) from each tree at a depth of 0–30 cm. Roots for the herbivory study were collected in 1994, and for the competition and parasitism study in 2002. We classified between 75 and 100 living EM root tips per tree based on morphology and stored the EM root tips in 1.5 ml microcentrifuge tubes at –20°C until molecular analyses were conducted. This level of sampling has been shown to adequately characterize the EMF community as extensive assessment of *P. edulis* showed that individual trees had seven or fewer species, with two species dominating (82%) the community (Gehring et al., 1998). We extracted the DNA from a minimum of two to three root tips of each morphotype from each tree using DNeasy Kits (Qiagen, Valencia, CA, USA). We used the mini-prep method of (Gardes and Bruns, 1993) to extract DNA from the herbivory samples collected in 1994. DNA extraction and amplification success was similar for samples collected during all years, averaging >90%. We amplified the internal transcribed spacer (ITS) region of the fungal genome, located between the 18S and 28S rRNA, using PCR (polymerase chain reaction) with the ITS1F and ITS4 primer pair (Gardes and Bruns, 1993). Morphotypes were characterized by a single species of EMF, except for the smooth, red-brown morphotype that characterizes the genus *Geopora*. Multiple closely related species of *Geopora* are found on *P. edulis* (Gordon and Gehring, 2011); additional sequencing was done to estimate the relative abundance of the *Geopora* species if multiple species were found in the initial screening. We assembled forward and reverse DNA sequences in BioEdit version 7.0.5.3 (Hall, 1999) to create a consensus sequence that was used in a BLASTn search on the NCBI and UNITE websites (Altschul et al., 1990; Abarenkov et al., 2010). We used percentage query coverage, percentage maximum identity, and bit score data to identify the closest match of our fungi to those in these databases. The names of some species reported in previous papers were modified based on cross-referenced nomenclature and phylogenetic placements with Index Fungorum (http://www.indexfungorum.org) accessed during January of 2014.

We visualized data on the community composition of EMF associated with the six groups of trees using relative abundance data (the percentage of a given EMF species relative to all EMF root tips in a sample) and non-metric multidimensional scaling (NMS) ordinations with a Bray-Curtis distance measure in PC-ORD 5.10 (McCune and Mefford, 2006). We used an analysis of similarity (ANOSIM) in PRIMER version 6.1 (Clarke and Gorley, 2006) to determine if the EMF communities of low biotic interaction trees (low herbivory, low competition, low parasitism) differed from one another. We used the same type of analysis to determine how the EMF communities of high biotic interaction trees compared to one another. Hypothesis 1 would be supported if we observed significant differences among communities in low interaction trees, but no difference in community composition in high interaction trees.

### HYPOTHESIS 2: COMMUNITIES OF EMF WILL RESPOND SIMILARLY TO THE ABIOTIC STRESS OF DROUGHT AS THEY DO TO THE BIOTIC STRESS OF HERBIVORY

We addressed this hypothesis by re-sampling the juvenile trees that experienced high versus low levels of herbivory in 2004, ten years after the first sampling (*n* = 14 trees per group). The trees were still non-reproductive in 2004. The first year sampled, 1994, occurred at the end of a period of wet years, while the second, 2004, occurred during a period of ongoing drought. Average early year (January–May) precipitation totaled 188.4 mm for the 5 year before the 1994 collection and 86.6 mm for the 5 year before the 2004 collection (Sthultz et al., 2009a, b). The persistently dry conditions beginning in 1995–1996 resulted in extensive mortality of *P. edulis* in northern Arizona (Mueller et al., 2005). The methods used to characterize EMF communities were similar for the 2004 and 1994 sampling periods, with the exception of DNA extraction using the mini-prep method in 1994 as noted above. Likewise, community data were visualized using ordinations in PC-ORD. We tested the influence of insect herbivory (insect susceptible high versus insect resistant low) and year (1994 versus 2004) on EMF community composition with a permutation-based nonparametric multivariate analysis of variance (PerMANOVA; Anderson, 2001) using relative abundance data in PRIMER version 6.1 (Clarke and Gorley, 2006). We sampled the same trees each year and accounted for this repeated sampling by including tree identity as a factor nested within the insect resistance category. We analyzed the main effects of herbivory and year as a two-way factorial (*P* ≤ 0.05). Hypothesis 2 would be supported if the EMF communities of *P. edulis* experiencing low levels of herbivory shifted with drought to resemble those of *P. edulis* experiencing high levels of herbivory. We did not expect the community composition of trees experiencing high herbivory to change with drought.

### HYPOTHESIS 3: PLANTS COLONIZED BY THE EMF COMMUNITY ASSOCIATED WITH HIGH HERBIVORY, PARASITISM, AND COMPETITION WILL EXHIBIT POOR GROWTH

We tested this hypothesis by sampling EMF communities and shoot growth in an independent set of juvenile *P. edulis*. These trees had experienced chronic scale insect herbivory in the past, but these insects had been mechanically removed for 19 years, allowing both foliage and EMF abundance to completely recover (Cobb and Whitham, 1993; Gehring et al., 1997). Preliminary measurements indicated that these trees had EMF communities that encompassed those of both high and low herbivory, allowing us to examine the effects of community variation without the complication of variation in parasitism, competition, or foliage loss due to herbivory. We sampled fifteen susceptible trees that had had their insects experimentally removed for EMF communities in August 2004, using the methods described previously. At the same time, we measured the length of ten shoots per tree (2004 growth only) as an estimate of tree growth. We compared the relative abundance of three members of the genus *Geopora* with shoot growth using regression analysis in IBM SPSS version 20. We chose the relative abundance of these *Geopora* as our measure of EMF community variation because our tests of Hypothesis 1 indicated that these taxa increased substantially in association with parasitism, competition, and herbivory (see below). Hypothesis 3 would be supported if we observed a significant negative relationship between shoot growth and the abundance of *Geopora* in the EMF community.

## RESULTS

### HYPOTHESIS 1: COMPETITION, PARASITISM, AND HERBIVORY WILL HAVE SIMILAR EFFECTS ON EMF COMMUNITY COMPOSITION

**FIGURE 1:**
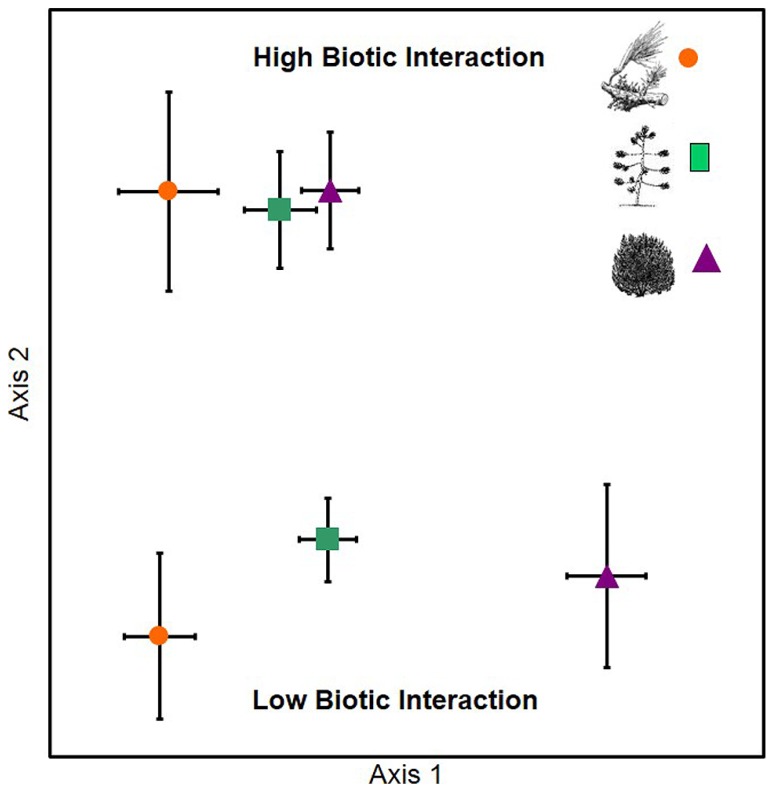
**An NMS ordination showing that pinyon pines that experienced low levels of plant parasitism, herbivory or competition differed significantly in EMF community composition from one another (bottom portion of graph) while trees with high levels of parasitism, herbivory or competition had very similar EMF communities (top portion of graph)**. Data represent the community centroids and the SE surrounding those centroids as follows: orange circles – mistletoe parasitism; green squares – scale insect herbivory; purple triangles – competition with juniper.

In support of hypothesis 1, the EMF communities associated with *P. edulis* experiencing low levels of parasitism, herbivory, and competition were significantly different from one another (*A* = 0.172, *P* < 0.001), while the communities of *P. edulis* experiencing high levels of these same interactions were similar (*A* = 0.015, *P* = 0.651; **Figure 1**). We observed 18 species of EMF across the six groups of trees; members of the genera *Geopora* (five species) and *Rhizopogon* (three species) were the most common but we also observed species in the genera *Tricholoma, Lactarius, Inocybe, Russula, Cortinarius*, and *Tomentella.*

**FIGURE 2:**
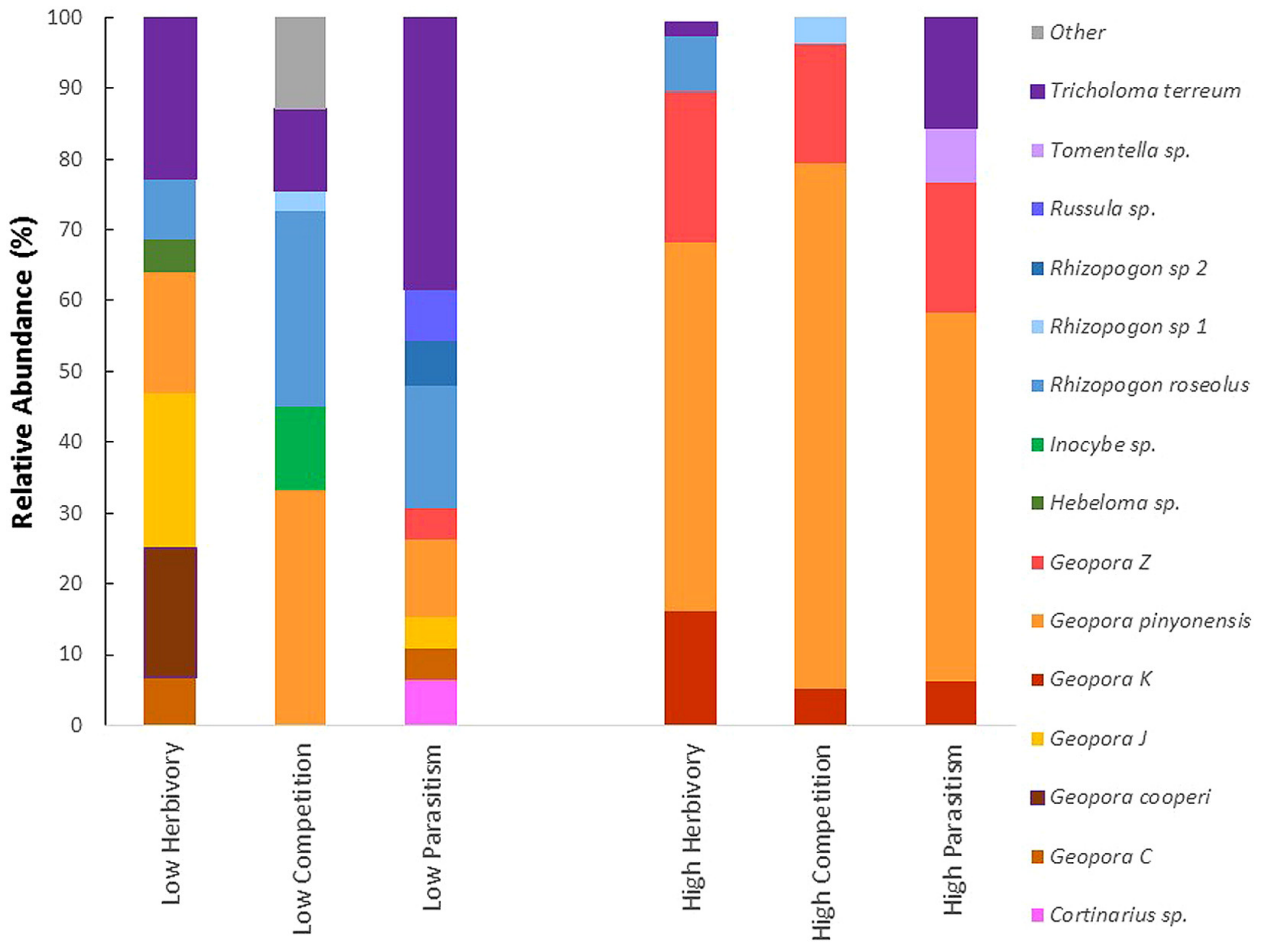
**The relative abundance of fifteen taxa of EMF observed in association with *Pinus edulis* experiencing high (susceptible) versus low (resistant) insect herbivory, plant parasitism or competition with *Juniperus monosperma***. Only taxa making up 3% or more of the community of a tree were included on the graph. The communities of *P. edulis* experiencing high levels of biotic interactions are dominated by the same three members of the genus *Geopora*. The gray portion of the bar labeled “other” groups species that were found in 3% or lower relative abundance.

All three of the high biotic interaction communities were dominated by the same three members of the genus *Geopora* that made up 95, 89, and 77% of the relative abundance in the high parasitism, high competition, and high herbivory trees, respectively (**Figure 2**). Members of this genus were much less common on low biotic interaction trees, averaging 39% relative abundance. Among the low interaction trees, the relative abundance of *Geopora* was highest on low herbivory trees. However, most of the *Geopora* observed on these trees were of different species than the *Geopora* observed on high biotic interaction trees, and included *G. cooperi*, which appears to be phylogenetically distinct from the other species (**Figure 2**; Guevara-Guerrero et al., 2011; Stielow et al., 2012; Flores-Rentería et al., 2014). Members of the genus *Rhizopogon* dominated *P. edulis* experiencing low competition, while *Tricholoma terreum* dominated *P. edulis* experiencing low parasitism (**Figure 2**).

### HYPOTHESIS 2: COMMUNITIES OF EMF WILL RESPOND SIMILARLY TO THE ABIOTIC STRESS OF DROUGHT AS TO THE BIOTIC STRESS OF HERBIVORY

**FIGURE 3:**
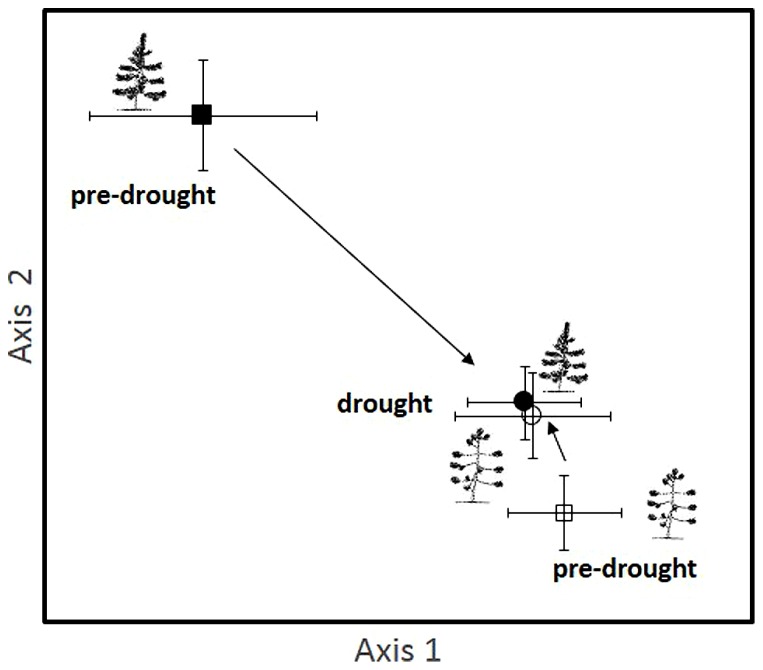
**An NMS ordination showing the EMF communities of high insect herbivory (susceptible trees) and low insect herbivory (resistant) trees during a pre-drought period in 1994 and a drought period that began in 1996 and continues to the present**. The tree types are represented by different symbols (open symbols indicate susceptible, high herbivory, which is also indicated by the icon showing the poodle tail architecture resulting from high foliage loss; closed symbols indicate resistant, low herbivory shown by an icon with a full complement of needles). Pre-drought samples are indicated with squares and drought samples with circles. Each point represents the centroid of the EMF community of 14 replicates per treatment with vertical and horizontal bars depicting ±1 SE. Arrows show trajectories of communities from the pre-drought to the drought period.

Our hypothesis that drought would result in similar shifts in EMF community composition as insect herbivory was supported. The EMF communities of susceptible, high herbivory trees were similar in pre-drought and drought years, while the EMF communities of resistant, low herbivory trees changed substantially during the drought year, becoming more like the communities of high herbivory trees (**Figure 3**).

This change in low herbivory trees was supported by a significant herbivory by year interaction across 10 years from normal to severe drought conditions (Pseudo *F*_1__,53_ = 2.52, *P* = 0.041). The main effect of herbivory was also statistically significant (Pseudo *F*_1__,53_ = 2.86, *P* = 0.014), while the main effect of year was not statistically significant (Pseudo *F*_1__,53_ = 1.902, *P* = 0.109). The three members of the genus *Geopora* observed to increase dramatically with herbivory, competition, and parasitism also increased substantially during the drought year in low herbivory trees, shifting from 16% of the community to 58% of the community. We sampled the same trees for EMF communities in both 1994 and 2004, but tree identity did not explain a significant portion of the variation in community composition (Pseudo *F*_25,53_ = 0.747, *P* = 0.936).

### HYPOTHESIS 3: PLANTS COLONIZED BY THE EMF COMMUNITY ASSOCIATED WITH HIGH HERBIVORY, PARASITISM, AND COMPETITION WILL EXHIBIT POOR GROWTH

**FIGURE 4:**
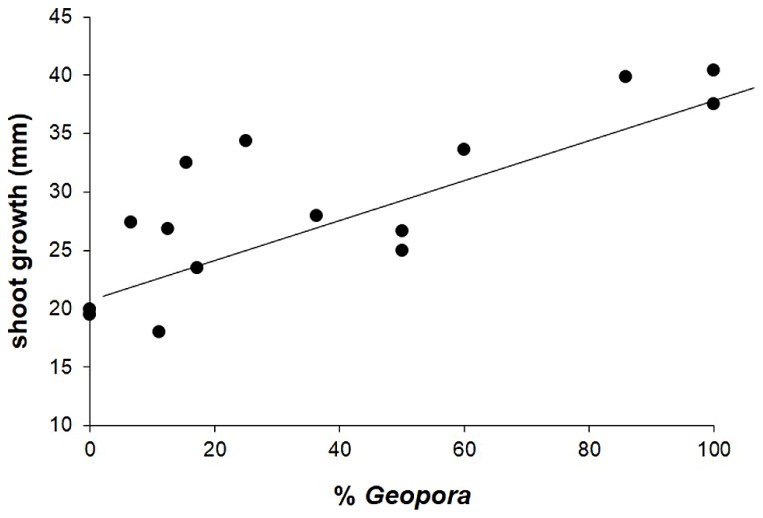
**Shoot growth of juvenile *Pinus edulis* trees in the field was significantly, positively associated with the relative abundance of three common members of the genus *Geopora***. See text for statistics.

In contrast to our hypothesis, shoot growth was significantly positively correlated with the abundance of the three species of *Geopora* that dominated on trees that experienced high levels of competition, parasitism, and herbivory (*r*^2^ = 0.574, *F*_1,13_ = 17.454, *P* = 0.001; **Figure 4**).

## DISCUSSION

### COMMUNITY CONVERGENCE TOWARDS GENERALIST ECTOMYCORRHIZAS

The convergence of EMF communities in response to biotic and abiotic stressors is consistent with several of the predictions of Chase (2003) who argued that community assembly would lead to a single equilibrium state in environments with small regional species pools, high dispersal potential, low levels of productivity and frequent disturbance. Relative to better studied plant communities, fungal communities assemble and disassemble rapidly, and are likely more linked to finer-scale environmental changes, which helps explain why the communities of trees experiencing low competition, herbivory, and parasitism were different, while trees under abiotic or biotic environmental stress (e.g., disturbance), were not. Chase (2003) also found that as site productivity increased, communities at the same site became more dissimilar. Consistent with these results, *P. edulis* experiencing low levels of drought and/or negative biotic interactions likely represented high productivity environments for EMF, promoting community dissimilarity. Ikeda et al. (2014) used similar arguments to predict that changes in host productivity with climate change would influence the community structure of dependent communities such as mycorrhizal mutualists and herbivores.

The EMF communities of *P. edulis* experiencing high biotic and abiotic stress converged toward a community highly dominated by three species within the same genus, *Geopora*. A review of the effects of past and current climate change on species interactions indicated that climate change frequently resulted in communities dominated by generalist species and interactions (Blois et al., 2013). The distribution and symbiotic traits of members of the genus *Geopora* are poorly understood, yet they appear to be generalists. They have been observed on both gymnosperm and angiosperm hosts (Fujimura et al., 2005; Hrynkiewicz et al., 2009; McDonald et al., 2010), and in association with ecosystems ranging from arid shrubland to boreal forest (Tedersoo et al., 2006; McDonald et al., 2010). Members of the genus *Geopora* were the principal EMF colonists of willow clones planted for restoration in fly ash that had been inoculated with another genus of EMF (Hrynkiewicz et al., 2009), suggesting they may disperse readily and survive well in harsh environmental conditions. Previous studies with *P. edulis* also documented increases in the relative abundance of members of this genus within and among sites as drought intensified in the southwestern United States (Sthultz et al., 2009a, b; Gordon and Gehring, 2011; Gehring et al., 2014). Interestingly, convergence toward *Geopora* dominance happened more rapidly with drought in the scale resistant juvenile *P. edulis* described here than in moth resistant mature *P. edulis* at the same site (Gehring et al., 2014). Given that all of the *P. edulis* studies described patterns of EMF communities before and during drought, thereby confounding drought and sampling time, alternative explanations for the community shifts are possible. Experimental work is necessary to substantiate these patterns and to explore mechanisms.

The community convergence observed across the three biotic interactions is striking given that shifts in abundance, measured as percent root colonization, were positive in some studies, but not in others. Abundance of EMF was lower on pinyons with high levels of root competition and insect herbivory, but was higher on trees with high plant parasitism (Haskins and Gehring, 2004; Mueller and Gehring, 2006). This finding suggests that even when pinyon hosts invested more in the EMF symbiosis following parasitism, they tended to associate with a limited group of EMF. The extreme convergence we observed is also surprising given that the site where plant parasitism was studied was more than 30 km distant from the others, with distinct soil characteristics, particularly soil nutrients. We would have expected a different pool of EMF to be present in this site, including a different subset of species tolerant of high biotic stress. As mentioned above, we know little about the biology of members of the genus *Geopora* that would help explain these patterns. However, relatives of the genus *Geopora* in the order Pezizales were reported to have significant saprotrophic abilities (Tedersoo et al., 2010), which could allow them to persist in situations when they are poor competitors with other EMF for root colonization sites.

### ECTOMYCORRHIZAL FUNGAL COMMUNITY DISASSEMBLY

The species losses and community convergence of EMF we observed in response to multiple environmental stressors is indicative of community disassembly. Community disassembly has been observed in response to global changes such as habitat destruction and climate change (reviewed in Zavaleta et al., 2009), and can occur over very short time scales, particularly with environmental perturbations that alter species interactions, such as invasion by an exotic species (Sanders et al., 2003). In many studies that observed community disassembly, species losses were associated with specific traits, such as rarity or degree of specialization (Zavaleta et al., 2009). For organisms involved in symbioses, traits that directly or indirectly impact the fitness of their partner may also impact their own survival, particularly under stressful conditions. Here we documented how multiple biotic and abiotic stressors acted in concert to favor a community of generalist ectomycorrhizal fungal mutualists. These seemingly disparate drivers of community disassembly may have had similar effects on EMF communities because they altered the trading relationships within the symbiosis, favoring fungi with low carbon demands as the photosynthetic capabilities of the host were compromised. The carbon demands of *Geopora* relative to other EMF have not been studied, but they have the morphological characteristics described for low cost fungi in other systems (Saikkonen et al., 1999).

Although the ectomycorrhizal symbiosis is generally considered mutualistic, it can be constructive to think of mutualisms in the context of reciprocal cheating, which persists only when both partners are able to prevent cheating by the other (Hoeksema and Kummel, 2003). Shifting abiotic conditions can alter the impact of biotic interactions (Agrawal et al., 2007). The cost to benefit ratio of the ectomycorrhizal symbiosis has been shown to change under different environmental conditions (Kennedy and Peay, 2007), and host plants have been shown to regulate their EMF partners under changing environmental conditions. For example, Peay et al. (2010) found that seedlings were able to maintain high growth rates under experimental nutrient enrichment by reducing colonization by EMF of the genus *Rhizopogon*. Across a natural environmental gradient, Moeller et al. (2013) found that the traits of EMF reflected the nutritional needs of their host plants, with communities composed of efficient foragers with high carbon requirements dominating in nutrient deficient soils. Because *Geopora* is a common member of the EMF communities found on pinyons, trees on which less efficient mutualists were eliminated were able to maintain higher growth rates as in Peay et al. (2010). The strong positive relationship observed between dominance by *Geopora* and pinyon growth suggests that although community disassembly was often considered detrimental (Zavaleta et al., 2009), negative effects may not always be observed, at least in the short term. The abundance of *Geopora* also was positively associated with host plant growth in another study of trees that experienced drought for a longer period than our study trees (Gehring et al., 2014). In addition, *P. edulis* that survived extreme drought were dominated by members of this EMF community (Swaty et al., 2004; Sthultz et al., 2009a, b). Taken together, these studies suggest that community disassembly may be a critical response to stress that favors the host tree and a subset of the EMF community.

### LONG-TERM EFFECTS OF EMF COMMUNITY CONVERGENCE

In a drought year, pinyons colonized by EMF communities dominated by *Geopora* had higher growth rates, but the long-term effects of hosting such constrained communities are unclear. Plants have been shown to benefit from hosting a highly diverse EMF community (Baxter and Dighton, 2001; Jonsson et al., 2001), likely because this results in higher functional diversity of EMF traits, such as the ability to access different forms of phosphorus and nitrogen (Baxter and Dighton, 2005). However, whether communities composed of closely related species have lower functional diversity is unclear. Studies linking community relatedness and functional diversity have found both negative (Burns and Strauss, 2011) and positive relationships (Prinzing et al., 2008). For EMF, this could be further complicated by studies that showed that the relative effects of EMF species on host growth can change depending upon environmental conditions (Kipfer et al., 2012). As a result, it is possible that communities composed primarily of *Geopora* could be less beneficial under more benign environmental conditions.

Community convergence could potentially alter the relative cost to benefit ratio of EMF communities dominated by *Geopora* under non-drought conditions, but another possible outcome is the loss of biodiversity within the larger EMF community. Arid conditions are predicted for the duration of this century in the southwestern USA (Seager et al., 2007), and concurrent increases in herbivory and competition that can result from warmer, drier conditions (Anderegg et al., 2013) could facilitate the persistence of *Geopora*-dominated communities to the detriment of other species of EMF. In the high stress situations we observed, the relative abundance of three common species of *Geopora* averaged 87%. An additional site in which *Geopora* was uncommon in association with *P. edulis* shifted to *Geopora* dominance with drought (Gordon and Gehring, 2011). Ongoing drought could lead to the extirpation of once common species of EMF from large areas of northern Arizona. The persistence of these formerly common species may rely on their survival as propagules in the soil, a poorly understood aspect of the biology of EMF. Data from the most comprehensive study of spore longevity in EMF to date showed that several initially abundant species persisted for a minimum of six years as spores, while other, initially less common species were no longer observed after the same time period (Nguyen et al., 2012). Locally extirpated species of EMF could also colonize from areas more favorable for their growth and reproduction. However, long distance dispersal may be required as recent studies suggest that EMF propagules decreased rapidly with increasing distance from spore sources (Peay et al., 2012).

## CONCLUSION

Several conclusions have emerged from our long-term studies spanning wet to record dry conditions. First, diverse stressors including plant parasites, insect herbivores, competing trees, and drought similarly altered the EMF communities associated with an iconic foundation tree species that characterizes much of the arid American Southwest. Second, this community disassembly resulted in convergence towards a few closely related, generalist species of EMF. Third, while this community shift had negative consequences for the distribution of previously dominant fungi, the change may be beneficial for host plants because the remaining EMF community members were better mutualists under current, drought conditions. Fourth, the long-term trajectory of community disassembly appeared to follow some of the “rules” of community disassembly observed in other systems, demonstrating the importance of both the drivers of change and the abiotic context in which they were found.

## AUTHOR CONTRIBUTIONS

Catherine A. Gehring, Rebecca C. Mueller, Kristin E. Haskins, Tine K. Rubow, and Thomas G. Whitham designed and conducted the initial studies upon which the synthesis in this manuscript was based. Tine K. Rubow and Catherine A. Gehring conducted the subsequent sampling of a subset of the trees during drought. Catherine A. Gehring analyzed the data and wrote the first draft of the manuscript. Rebecca C. Mueller, Kristin E. Haskins, and Thomas G. Whitham provided valuable comments on the manuscript. Tine K. Rubow passed away before the first draft of the manuscript was written.

## Conflict of Interest Statement

The authors declare that the research was conducted in the absence of any commercial or financial relationships that could be construed as a potential conflict of interest.
